# Antioxidant, Anti-Inflammatory and Antidiabetic Proprieties of LC-MS/MS Identified Polyphenols from Coriander Seeds

**DOI:** 10.3390/molecules26020487

**Published:** 2021-01-18

**Authors:** Hamza Mechchate, Imane Es-safi, Amal Amaghnouje, Smahane Boukhira, Amal A. Alotaibi, Mohammed Al-zharani, Fahd A. Nasr, Omar M. Noman, Raffaele Conte, El Hamsas El Youbi Amal, Hicham Bekkari, Dalila Bousta

**Affiliations:** 1 Laboratory of Biotechnology, Environment, Agrifood, and Health, University of Sidi Mohamed Ben Abdellah, FSDM-Fez 47963, Morocco; imane.essafi1@usmba.ac.ma (I.E.-s.); amal.amaghnouje@usmba.ac.ma (A.A.); smahaneboukhira@gmail.com (S.B.); hicham.bekkari@usmba.ac.ma (H.B.); dalila.bousta@usmba.ac.ma (D.B.); 2 Basic Science Department, College of Medicine, Princess Nourah bint Abdulrahman University, Riyadh 11671, Saudi Arabia; amaalotaibi@pnu.edu.sa; 3 Biology Department, College of Science, Imam Mohammad ibn Saud Islamic University (IMSIU), Riyadh 11623, Saudi Arabia; mmyalzahrani@imamu.edu.sa; 4 Medicinal Aromatic, and Poisonous Plants Research Centre, College of Pharmacy, King Saud University, Riyadh 11451, Saudi Arabia; fnasr@ksu.edu.sa (F.A.N.); onoman@ksu.edu.sa (O.M.N.); 5 Research Institute on Terrestrial Ecosystems (IRET)—CNR, Via Pietro Castellino 111, 80131 Naples, Italy; raffaele.conte86@tiscali.it; 6 Laboratory of Biology and Health, Faculty of Sciences Ben M’Sik, Hassan II-Casablanca University, Casablanca 20000, Morocco; amal.elyoubi@gmail.com

**Keywords:** *Coriandrum sativum* L., polyphenols, antioxidant propriety, anti-inflammatory, antidiabetic, antihyperlipidemic, antihyperglycemic, DPPH, β-carotene discoloration

## Abstract

*Coriandrum sativum* L. seeds are traditionally used to treat diabetes and its complications (inflammation and formation of reactive oxygen species) around the world. The present study investigates the antidiabetic, anti-inflammatory, and antioxidant effects of the polyphenol fraction of *Coriandrum sativum* seeds (PCS). Diabetic mice were orally administered with PCS (25 and 50 mg/kg b.w.) for 28 days. Oral glucose tolerance (OGTT) was also evaluated along with the anti-inflammatory effect, assessed by measuring paw edema development induced with carrageenan in Wistar rat and the antioxidant activity assessed using two tests (β-carotene discoloration and DPPH). Treatment of diabetic mice with PCS for four weeks managed their high fasting blood glucose levels, improved their overall health, also revealed an excellent antihyperlipidemic activity. The OGTT result showed a potent antihyperglycemic activity, and following the anti-inflammatory and antioxidant effects, the PCS exhibited a perfect activity. LC-MS/MS result revealed the presence of 9 polyphenols. This modest work indicates that the PCS have an important antidiabetic, antihyperglycemic, antihyperlipidemic, anti-inflammatory, and antioxidant effect that can be well established treatment of diabetes and its complications.

## 1. Introduction

Diabetes refers to a category of disorders that affect the way our body uses glucose in the blood. It threatens millions of people and has a remarkably high prevalence [1]. About 693 million cases were predicted by the International Diabetes Federation (IDF) in 2045, compared with just 151 million cases in 2000 [2]. After the hyperglycemia manifestation, diabetes complications begin with serious changes in the lipid’s metabolism [3], oxidative stress, and inflammation [4]. Oxidative stress occurring during the diabetic state results from the auto-oxidation of glucose and the production of free radicals and the lack of antioxidants in the body, leading to the development of inflammations, apoptosis, and multiple health problems [5]. Throughout the years, chemical and synthetic antidiabetic medications have been developed to help repair certain forms of dysfunctions that control the diabetic condition and avoid complications (hypoglycemia, hypertension, and many others) [6]. Still, their extended use’s detrimental effects have diverted many researchers’ focus on seeking a new alternative [7].

New studies are now devoted to medicinal plants and natural compounds that may have positive effects on diabetes [8]. Thus the use of medicinal plant-derived compounds has a multi-effect on glycemic and lipid profiles, oxidative stress, and inflammation may improve the management of diabetes multiple complications [9]. Medicinal plants have been traditionally used as alternative remedies, playing a major role in new pharmaceutical drugs production with a safe and effective profile [10]. They have gained high attention lately with increased studies and research confirming their therapeutic proprieties [11]. Researchers over the years have approved the antidiabetic potential of medicinal plants such as *Thymus satureioides* [12], *Ammodaucus leucothricus* [13] and many others this potential when it deeply studied was attributed mostly to the molecule family of flavonoids [14] and polyphenols [15]. Among the medicinal plant with current interest appears coriander (*Coriandrum sativum* L.; Umbelliferae), known locally as “Kassbour,” is widely distributed and mainly cultivated for the seeds [16]. Coriander seed is a common spice that has enormous documented traditional medicinal use as a treatment of diabetes, gastrointestinal disorders, kidney and heart problems [7,17,18,19,20] and also for fighting worms, rheumatism, and joint pain [16]. Coriander whole seeds (more than 20% oil and many different components) were being studied for their hypolipidemic effect [21], its role in decreasing serum glucose and increased insulin release [22] and the oil part was studied for its radical scavenging activity [23] and anti-inflammatory activity [24]. As the most explored part of the seeds were its oils rich on linalool, geranyl acetate, p-cymene, camphor and caryophyllene which have many uses in food, cosmetic and also health industries [25]. This work’s main focus is highlight and reveal the hidden health benefit of the polar part of the coriander seeds usually been discarded by determining it active molecules (via LC-MS/MS) and their antidiabetic, antihyperglycemic, antihyperlipidemic, anti-inflammatory, and antioxidant proprieties. 

## 2. Results and Discussion

### 2.1. PCS Qualitative Analysis

**Figure 1:**
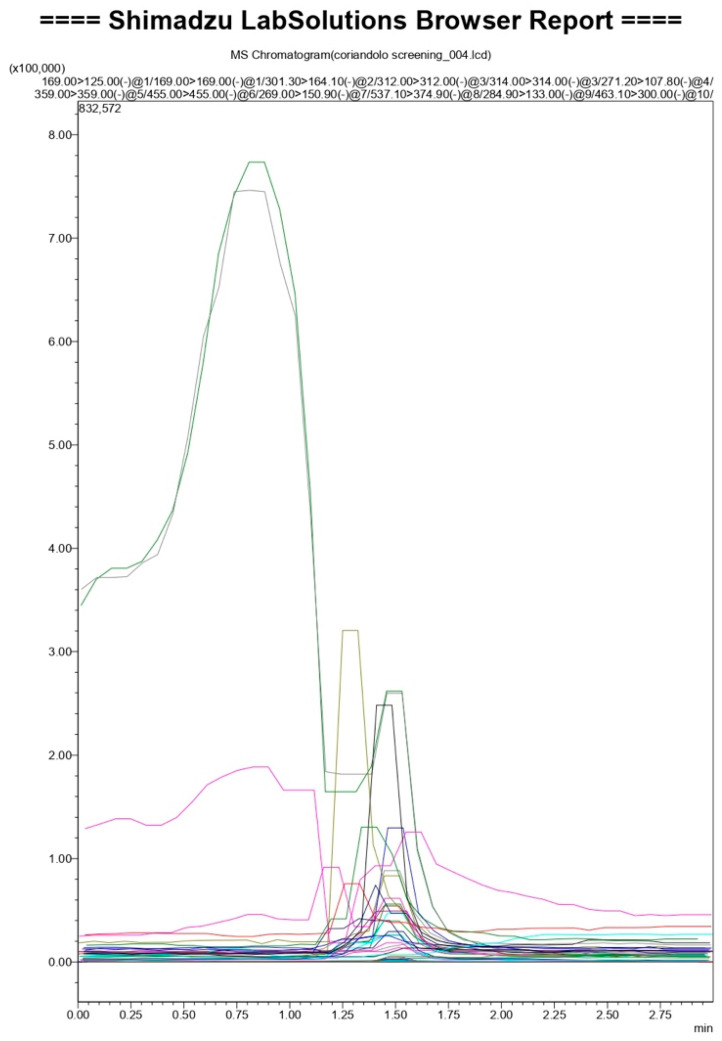
Selected fragments derive from the molecular weight of the polyphenol minus one (due to ESI- ionization) and their subsequent fragmentation in MS/MS experiment. Notes: Vanillic acid in black, Chlorogenic acid in violet, Catechin/Epicathechin in dark blue, Oleuropein in dark green, Epicathechin gallate in brown, Rutin in light green, Gallocathechin/Epigallocathechin in light blue.

**Figure 2:**
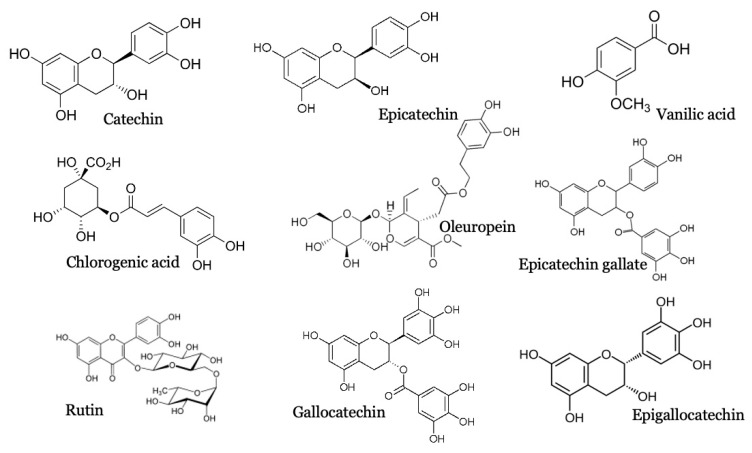
2D structure of PCS-identified molecules.

Figure 1 and Table 1 represent the LC-MS/MS result. PCS analytes have very close retention times. However, accurate identification was made according to their typical fragments and through comparison with standards available in the developed database. The results indicated that PCS comprises nine molecules belonging to polyphenols (Vanillic acid: C8H8O4, chlorogenic acid: C16H18O9, catechin: C15H14O6, epicatechin: C15H14O6, oleuropein: C25H32O13, epicatechin gallate: C22H18O10, rutin: C27H30O16, gallocatechin: C15H14O7, epigallocatechin: C22H18O11); five of them belong to the flavonoid family, which make this extract a flavonoids-rich extract (Figure 2).

Some of these molecules are studied for their antidiabetic activity (Rutin) [26] anti-inflammatory (Vanilic acid, chlorongenic acid) [27,28] and antioxidant activity (Catechins) [29,30]. Their synergetic effect has been studied in this work. 

### 2.2. Evaluation of the Antidiabetic Activity

#### 2.2.1. PCS Effects on FBG after Subacute Administration

**Figure 3:**
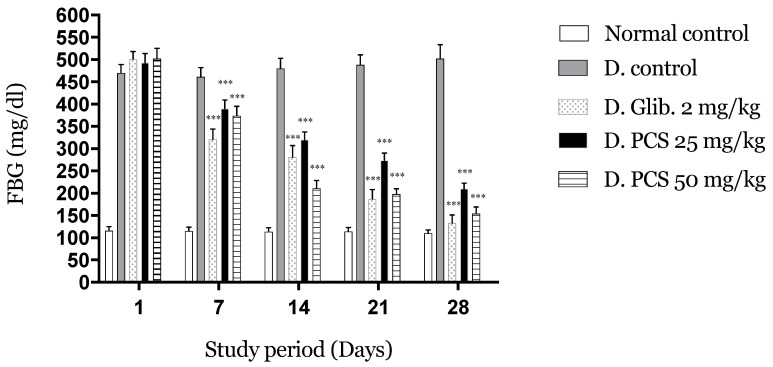
Four-week administration of PCS effect on fasting blood glucose. Values are expressed as mean ± SD (*n* = 5 mice). *** *p* < 0.001 compared to diabetic control.

Figure 3 shows the effects of repeated oral administration of PCS at different doses. 

After the first week, the reduction reached 20% for PCS at the dose of 25 and 25% for the dose of 50. After that, diabetic condition and hyperglycemia were successfully controlled indicated by a significant reduction (*p* < 0.001) (Day 14: 35,58,44%; Day 21: 44,61,63%; Day 28: 57,65,73% for doses 25, 50 and, glibenclamide, respectively) compared to the negative control.

The 4-week administration of PCS to diabetic mice caused a remarkable decrease in FBG compared to the control groups.

Table 2 indicates the body weight changes of the various experimental groups. For four weeks, diabetic groups treated with PCS demonstrated a significant increase (*p* < 0.001) in bodyweight when comparing it to the diabetic control group, which exhibited a substantial decrease. A high blood glucose level is due to the lack of insulin. The kidneys work to get rid of the excess glucose and as a result weight loss occurs in this process (because of the dehydration and the calories loss) [31]. Another piece of research attributed the weight loss to the extreme catabolism of structural proteins and fats used instead of carbohydrates (unable to be used by the body) as an energy source [32], This revealed the critical role of insulin in the regulation of metabolism and catabolism of different key players in the development of diabetes and management of the overall blood glucose level [33].

Table 2 shows the effect of the prolonged administration of PCS (25 and 50 mg/kg b. w.) on ASAT and ALAT serum levels. A decrease was noted for the groups treated with PCS when compared to the diabetic control group (*p* < 0.001), which exhibited a significant elevation in the activities of those enzymes compared to the normal control group (*p* < 0.001). This elevation is attributed to a loss of functional integrity of the hepatocellular membranes and a cellular leakage. 

The liver enzymes, aspartate aminotransferase (ASAT), alanine aminotransferase (ALAT), are routinely used to evaluate liver function; they are considered markers of hepatocellular health [34]. The elevated level of these enzymes is a marker of liver pathology and might be attributed to Alloxan’s action [35].

Kidney markers (urea and creatinine) changes were also shown in Table 3. The results found that non treated diabetic mice displayed a significant (*p* < 0.001) increase in urea and creatinine as compared to normal control. On the other hand, the groups that received the PCS and glibenclamide significantly reduced their creatinine and urea levels in comparison to the diabetic group (*p <* 0.001). Their elevated level indicates a progressive renal damage [36] prevented by the administration of different treatments.

**Figure 4:**
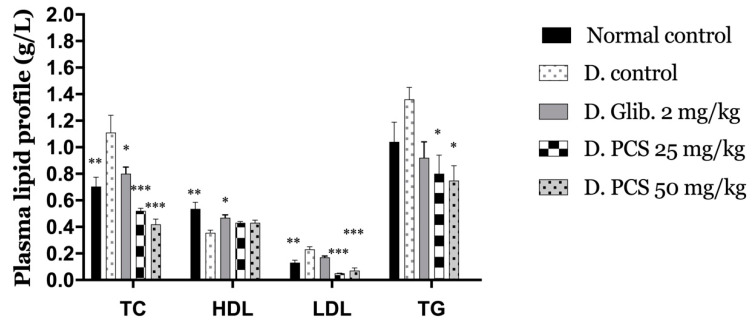
Effect of PCS on Plasma lipid profile in Alloxan-induced diabetic mice after 28 days of experiments. Values are expressed as mean ± SD (*n* = 5 mice). * *p* < 0.05, ** *p* < 0.01, *** *p* < 0.001, compared to diabetic control.

Figure 4 summarizes the plasma lipid profile of the experimental groups (triglycerides (TG), total cholesterol (TC), high-density lipoprotein (HDL), and low-density lipoprotein (LDL)). The levels of TC and TG were increased in the diabetic control group by +57% and +31%, respectively, in comparison to the normal control mice. This is in contrast to the other treatment, which showed a decrease compared to the diabetic control mice.

The administration of PCS to Alloxan-induced diabetic mice significantly (*p* < 0.001) reduced the level of LDL by 61% and 46% (25 and 50 b.w., respectively). At the same time, the glibenclamide group marked an increase in LDL level by 31% compared to normal control along with the diabetic control group, which has the most improvement with +77% compared to the normal control. As for The HDL level compared to normal control, all groups exhibited a decrease with −33% for diabetic control −12% noted for glibenclamide, and −20% for the groups treated with both doses of PCS. 

Dyslipidemia is associated with hyperglycemia, and both increase the prevalence of cardiovascular complications [37]. The characteristic features of diabetic dyslipidemia, as noticed in the diabetic control mice, are a high plasma level of triglyceride and LDL against low HDL concentration. The lipoprotein lipase enzyme, which hydrolyzes triglycerides, is activated by insulin. Its deficiency in the case of diabetes causes inactivation of this enzyme, resulting in hypercholesterolemia and hypertriglyceridemia [38], this results in an increase of LDL levels also leading to a decrease of HDL level [39]. As remarked, the alterations in serum lipid profile were attenuated after four weeks of administration of PCS to diabetic mice making the extract beneficial in improving lipid metabolism and by so preventing some diabetes complications.

#### 2.2.2. Oral Glucose Tolerance Test

**Figure 5:**
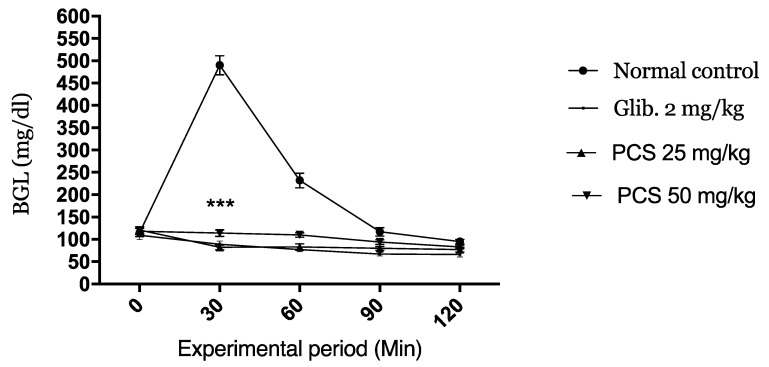
Effect of PCS on blood glucose level in Alloxan-induced diabetic mice during oral glucose tolerance (OGTT). Values are expressed as mean ± SD (*n* = 5 mice). *** *p* < 0.001compared to normal control.

The kinetic of the variation of the glucose level during the oral glucose tolerance (OGTT) test is represented in Figure 5. The normal control mice manifested a high blood glucose level 30 min after glucose load (495 mg/dL). Oral administration of PCS and glibenclamide induced a very significant decrease in blood glucose concentrations at 30 min (*p* < 0.001). The low BGL level were maintained at 60, 90, and 120 min when compared to the normal control groups.

Those postprandial blood glucose values indicate the excellent antihyperglycemic activity of the extract with both doses.

### 2.3. Anti-Inflammatory Activity

**Figure 6:**
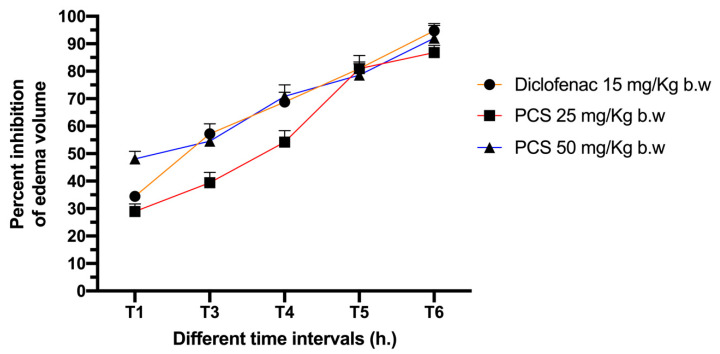
Effect of PCS on edema volume inhibition in carrageenan-induced paw edema Wistar rats.

A progressive edema formation is due to the injection of carrageenan into the rat’s hind paw, reaching its maximum within the first 60 min. The inhibition rate of the treatments is shown in Figure 6. Within the first hour, the inhibition rate reached 29% for the group of rats treated with PCS at 25 mg/kg b.w. and 48% for the group treated with the dose of 50 mg/kg b.w. against 34% marked for the groups treated with Diclofenac. The inhibition rate continues to rise to reach at the 3rd hour 39, 55, and 57% for groups treated with PCS at 25, 50 mg/kg b.w. and Diclofenac, respectively, those numbers set on a final inhibition rate of 87, 92, and 95% at the end of the test.

Those results clearly show the anti-inflammatory activity of PCS. This experiment is a standard experiment model to study acute inflammation, using carrageenan an approved chemical used to stimulate the release of inflammatory mediators. According to Vinegar et al. [40], carrageenan induces a biphasic response during edema formation. The first phase’s characterization is the release of, serotonin, kinins, and histamine in approximately the first hour after the injection. In contrast, the second phase is characterized by prostaglandins’ release in the next 2 to 3 h which are the main culprit responsible for acute inflammation [41]. PCS reveals a promoting anti-inflammatory effect as it exhibited a good activity in the first phase as well as the second phase of the inflammatory process.

### 2.4. Antioxidant Activity

Table 4 resumes the result of the overall antioxidant activity of the PCS.

#### 2.4.1. Scavenging of the Free Radical DPPH

Half-maximal inhibitory concentration (IC50) is the concentration needed to reduce half free radicals’ concentration. It is the measurement of the potency of a substance in inhibiting a specific biological or biochemical function.

According to the results, the IC50 for PCS was better than that of BHT(IC50 = 0.0005 ± 6.98 × 10^−6^ µg/mL) compared to BHT (IC50 = 0.14 ± 0.019 µg/mL).

#### 2.4.2. Discoloration Test of β-Carotene 

The test explores the capability of antioxidants to resist or postpone lipid oxidations in target molecules subjected to free radical sources. The measurement environment is near the actual lipid system in food products and humans [42]. According to the results described in Table 3, the oxidation inhibition for PCS was 53.73% against only 3.37% for the negative control.

For the evaluation of the antioxidant power of a given extract, a single method is not sufficient. Furthermore, different antioxidant assays with different principles and experimental conditions and necessary to validate the overall activity. The excellent antioxidant activity demonstrated by PCS could be attributed to its composition in polyphenols that have a high number of hydroxyl complexes and their known capacity to trap and neutralize free radicals [43]. 

## 3. Materials and Methods 

### 3.1. Plant Materiel

*Coriandrum sativum* L. seeds were obtained from a local herbalist. The taxonomical identification was made by Professor Amina Bari (Botanist), and a voucher specimen (BPRN28) was deposited in the herbarium of the LBEAS laboratory (USMBA, FSDM, Fez, Morocco).

### 3.2. Study Animal Selection 

For the study, mice (Swiss albino) (20–25 g) and rats (Wistar) (180–230 g) of both sexes were taken from the center of animals (FSDM). They were placed in suitable cages under controlled conditions (Humidity of 44–50% and a temperature of 23 ± 2 °C) and a light-dark cycle. Water and food were given ad libitum and the acclimatization period was set to 7 days. Animal were cared for, used and handled in total accordance with the internationally accepted standard guidelines [44] and the institution committee of animal ethics (02/2019/LBEAS).

### 3.3. Polyphenols Extraction 

Coriander seeds were well cleaned with tap water afterward dried and reduced to a fine powder. The first step was defatting the seeds (high content in oil); 10 g of powdered seeds were washed out with 30 mL hexane three times.

Extraction starts with methanol with an ultrasound-assisted extraction method (frequency set at 35 kHz). In total, 10 g of plant powder mixed with 100 mL of 70% methanol for 40 min and after that filtered using Whatman paper and concentrated utilizing a rotavap. The concentrated extract was rediluted in D.W. and extracted with n-butanol (repeated 2 times) to finally get the polyphenol (organic phase). 

### 3.4. Coriander Seed Polyphenol LC-MS/MS Analysis

Qualitative analysis of PCS was performed developing an in-house database comprising the secondary metabolites of polyphenols. In particular, mass fragments and mass spectra of phenolic compounds were obtained injecting the standards of each molecule in LC-MS/MS and identifying them according to their chromatographic separation on a Phenomenex Kinetex polar C18 column (3 × 100 mm^2^, 2.6 μm, Phenomenex, Torrance, USA) with mobile phase consisting of acetonitrile:water + 0.01% formic acid (5:95, *v/v*; isocratic). Such chromatographic separation was also confirmed by flow injection analysis mass identification based on the precursor ion’s detection and at least one characteristic fragment ion using a Shimadzu Ultra-High-Performance Liquid Chromatography (Nexera XR LC 40, Shimadzu Italy, Milan, Italy), coupled to an MS/MS detector (LCMS 8060, Shimadzu Italy, Milan, Italy) and controlled by Lab Solution software (Multi LC version) (see supplementary file). The MS/MS was operated in negative electrospray ionization (ESI-) setting the nebulizing gas flow at 3 L/min, heating gas flow 10 L/min, interface temperature 370 °C, DL temperature 250 °C, heat block temperature 450 °C, drying gas flow 10 L/min. Identification of PCS polyphenols, was performed suspending the coriander seeds fraction in 2 mL of water: acetonitrile 1:1. 20 μL of this solution was diluted in 980 μL of acetonitrile and injected for LC-MS/MS analysis.

### 3.5. Antidiabetic Activity Study

#### 3.5.1. Preparation and Induction of Experimental Diabetes 

The alloxan monohydrate solution needs to be freshly prepared just before the induction (in 0.9% cold saline without any contact of light). For the blood glucose level wanted to be induced in the animals the dose should be adjusted. in our study severe experimental diabetes was the objective, so the dose used of alloxan was 180 mg/kg given to the animals via an intraperitoneal injection after at least 12 h fast. In response to the alloxan rapid action (partial destruction of *β* cells) and liberation on their insulin content, a severe hypoglycemia was expected, so as a preventive action, the animals were given an oral administration of 0.2 mL of a glucose solution at 5 g/L. After 96 h and the stabilization of the animal’s blood glucose levels, first measurements were taken to select those with a BGL over 450 mg/dL for the test. 

#### 3.5.2. Groups Repartition and Experimental Model 

For this study, 5 groups were selected containing 5 mice each. The three first groups were controls (normal control group given 0.2 mL/day of D.W., negative diabetic control group given 0.2 mL/day of D.W., and a positive diabetic control group given 2 mg/kg/day of glibenclamide), and the last two groups were those treated with the extract (diabetic mice given PCS at 25 and 50 mg/kg/day).

The study period was set to 28 days (Subacute study) to observe the long-term effect of the extract on the diabetic state of the animals and also on their overall health.

The different treatments were given daily and orally by intra-gastric gavage. and the FBG was measured on day 1, 7, 14, 21, 28 using a glucometer (Accu-chek, Roche, Indianapolis, Indiana, USA). During those days, bodyweight measurements were also taken. On the last day of the study and after taking the usual measurements, the animals were anesthetized and sacrificed by cervical decapitation. A sample of the blood was collected to estimate biochemical parameters (Asat, Alat, Urea, Creatinine, HDL, LDL, TC, TG).

#### 3.5.3. The Oral Glucose Tolerance Test (OGTT)

In this experiment normal mice (5 in each group) were chosen to evaluate how PCS extract will manage high blood in a short time (2 h). Fasted animals were given orally the treatments and after 1 h they were given orally 5 g/kg of D-glucose to induce hyperglycemia.

The time when glucose was administered indicates the 0 times of the test. The next measurements were taken after 30, 60, 90, and 120 min.

### 3.6. Carrageenan Induced Paw Edema and Anti-Inflammatory Evaluation

Carrageenan was prepared at 1% in D.W. and injected (0.1 mL) into the rat’s right hind paw (sub plantar). As a result, an acute inflammation manifested as edema (acute inflammation) was induced [45].

The rats were divided into five groups, five rats each. Three first groups were controls (normal control group without edema induction given orally 2 mL of D.W., negative control group with induced edema given orally 2 mL of D.W., and positive control group given orally diclofenac at the dose of 15 mg/kg) and the last two groups were rats with induced edema given orally PCS at the doses of 25 and 50 mg/kg.

After 12h of food deprivation and 1 h before the starting of the test, all groups received their treatments. At “0” h, the paw thickness was measured before the injection and after 1, 2, 3, 4, and 6 h after. 

Anti-inflammatory activity was measured as the percentage reduction in edema level when the drug was present, relative to control.

### 3.7. In Vitro Antioxidant Activity

#### 3.7.1. DPPH Test

The free radical scavenging capability of PCS was checked by DPPH (2,2-diphenyl-1-picrylhydrazyl) assay, as demonstrated by Kedar and Singh [46]. The decoloration of the DPPH methanol solution determines the capacity of the hydrogen donation. DPPH (purple color) fades to yellow when antioxidants are present in the solution. Methanol was used to prepare a solution of 0.004% of DPPH (750 μL) and a sample solution (100 μL). Both prepared solutions were mixed and incubated at laboratory temperature for 30 min. Optic density was measured spectrophotometrically afterward at 517 nm. BHT was used as a positive and pure methanol as a negative control. The following equation determined the Percentage of DPPH radical scavenging activity:

(1) $$I(\%)=\left(\frac{A_{0}-A}{A_{0}}\right)\times 100$$

- *I*: inhibition Percentage.
- *A*_0_: Absorbance of the DPPH solution without a sample.
- *A*: Absorbance of the DPPH solution with the sample.

#### 3.7.2. β-carotene Bleaching Test

The *β*-carotene bleaching assay was performed according to the Prieto protocol [47]. In a vial, a solution containing 1 mL of a mixture of β-carotene and chloroform (1 mg/5 mL) was added to 100 μL of tween and 0.01 mL of linoleic acid. Evaporation of chloroform occurred after heating the mixture at 45 °C for 5 min. Then, 25 mL of oxygenated water (H2O2) was added to the residue. Next, 100 μL of each sample was diluted in methanol and added to 2.5 mL of the prepared mixture, then incubated in a water bath at 50 °C for 2 h. Optical density was measured at 470 nm. The blank contained the mixture solution without the β-carotene, and BHT was used as standard. The antioxidant activity was measured following this formula (Equation (2)):

(2) $$AA\%=\left(\frac{AE}{ABHT}\right)\times 100$$

- *AA*%: percentage of antioxidant activity.
- *AE*: absorbance after 2 h of the negative control.
- *ABHT*: absorbance after 2 h of BHT.

### 3.8. Statistical Analysis

Experimental data were presented as mean ± SD. Significant differences were compared by one-way ANOVA followed by Tukey post hoc test. Results were considered significant when *p* < 0.05. Graph Pad Prism (windows v7.0) was used to perform the statistical analysis 

## 4. Conclusions

This study confirms the traditional use of the seeds and also reveal the molecule family responsible for the activities. the polyphenol fraction of *Coriandrum sativum* L. seeds may serve as a treatment complement for both type 1 diabetes due to its anti-inflammatory and antioxidant activities and also for type 2 diabetes due antihyperglycemic propriety. PCS showed an overall remarkable antidiabetic, antihyperglycemic activity that may play a major role in managing diabetes and its complication. This research further encourages using this plant in conventional antidiabetic preparations and formulations and in a well-established antidiabetic phytomedicine or complement.

## Figures and Tables

**Table 1:** The detected molecules in the polyphenol fraction of Coriandrum sativum seeds (PCS) according to the LC/MS-MS results.

Molecule	Analyzed Fragment
Vanillic acid	167.00 > 123.00
Chlorogenic acid	353.00 > 190.00
Catechin\Epicathechin	289.00 > 179.00
Oleuropein	539.00 > 539.00
Epicathechin gallate	441.00 > 169.00
Rutin	609.00 > 301.00
Gallocathechin\ Epigallocathechin	305.00 > 125.00

**Table 2:** Bodyweight changes of the experimental groups during the subacute period of 28 days.

Treatment		Bodyweight Development	(g)		
	1st Day	7th Day	14th Day	21st Day	28th Day
Normal Control	23.4 ± 1.8	24.7 ± 1.5 *	25.2 ± 1.4 ***	26.9 ± 1.3 ***	27.2 ± 1.5 ***
Diabetic Control	23.8 ± 1.6	21.7 ± 2.2 ^#^	20.1 ± 2.7 ^###^	19.2 ± 2.5 ^###^	17.7 ± 2.4 ^###^
D. Glib 2 mg/kg	24.3 ± 1.6	23.1 ± 1.5	24.2 ± 1.7 **	25.8 ± 1.8 ***	25.9 ± 1.7 ***
D. PCS 25 mg/kg	22.5 ± 1.9	22.1 ± 1.6	23.6 ± 1.9 **	24.3 ± 1.9 **	26.2 ± 2.0 ***
D. PCS 50 mg/kg	21.9 ± 2.0	21.2 ± 1.9	22.34 ± 2.4 *	23.2 ± 2.6 **	24.8 ± 2.4 ***

Values are expressed as mean ± SD (*n* = 5 mice). ^#^ *p* < 0.05, ^###^ *p* < 0.001 compared to normal control; * *p* < 0.05, ** *p* < 0.01, *** *p* < 0.001 compared to diabetic control.

**Table 3:** Biochemical parameters of the experimental groups at the end of the study period.

	Liver Biomarkers		Kidney Biomarkers	
Treatments	ASAT(UI/L)	ALAT(UI/L)	Urea(g/L)	Creatinine(mg/L)
Normal control	45.8 ± 6.1	311 ± 21.2	0.28 ± 0.03	3.4 ± 0.54
Diabetic control	134 ± 12.2 ^###^	802 ± 98.3 ^###^	0.63 ± 0.05 ^###^	6.0 ± 0.62 ^###^
D. Glib 2 mg/kg	48 ± 4.4 ^***^	222 ± 15.2 ^***^	0.28 ± 0.03 ^***^	4.2 ± 0.35 ^**^
D. PCS 25 mg/kg	32 ± 3.2 ^***^	224 ± 15.6 ^***^	0.29 ± 0.02 ^***^	3.4 ± 0.30 ^***^
D. PCS 50 mg/kg	32 ± 2.9 ^***^	191 ± 13.2 ^***^	0.34 ± 0.02 ^***^	3.5 ± 0.36 ^***^

Values are expressed as mean ± SD (*n* = 5 mice). ^###^ *p* < 0.001 compared to normal control ** *p* < 0.01, *** *p* < 0.001 compared to diabetic control.

**Table 4:** Summary of in vitro antioxidant activity results.

	DPPH (IC50) (µg/mL)	β-carotene (Inhibition) (%)
PCS	0.0005 ± 6.98 × 10^−6^	53.73%
Positive control (BHT)	0.14 ± 0.019	_____
Negative control	_____	3.37%

Values are expressed as mean ± SD (*n* = 5).

## Data Availability

The data that support the findings of this study are available from the corresponding author, upon reasonable request.

## Supplementary Materials

The following are available online: Table S1: CSP Identified fragments according to the LC/MS-MS results.
