# Supplementary material for: Antioxidant, Anti-Inflammatory and Antidiabetic Proprieties of LC-MS/MS Identified Polyphenols from Coriander Seeds
Source: Molecules. 2021 Jan 18;26(2):487. doi: 10.3390/molecules26020487 (PMC7831938; doi:10.3390/molecules26020487)
Supplement: Supplementary file 1 [file molecules-26-00487-s001.pdf]

Molecule	Identified fragments	*Coriandrium sativum* seeds (PCS)	Standard in ACN (dil 1:1000)
Vanillic acid	167.00>123.00<br>167.00>167.00	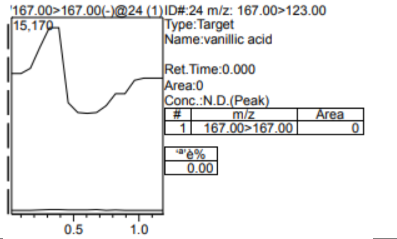	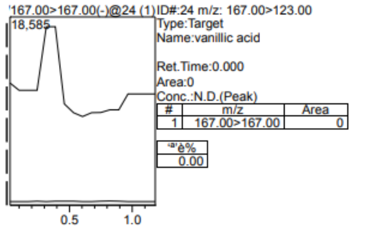
Chlorogenic acid	353.00>190.00<br>353.00>353.00	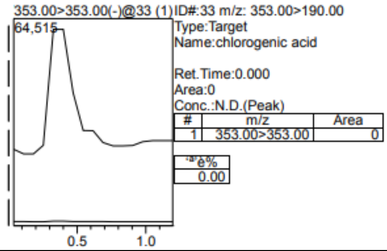	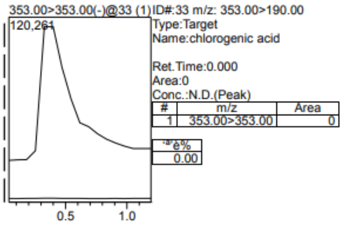
Oleuropein	539.00>539.00	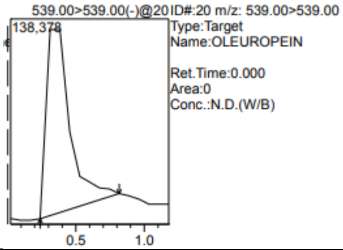	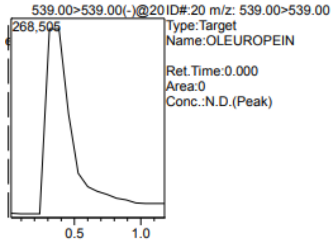
Epicatechin gallate	441.00>289.00<br>441.00>441.00	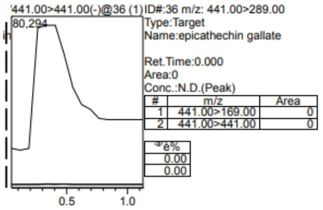	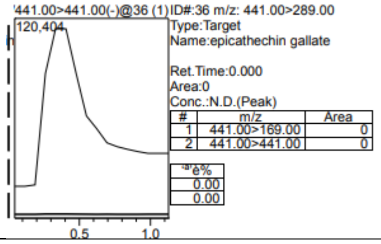
Catechin\ Epicatechin	289.00>245.00<br>289.00>289.00	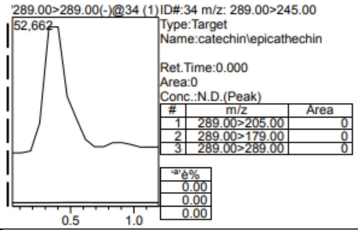	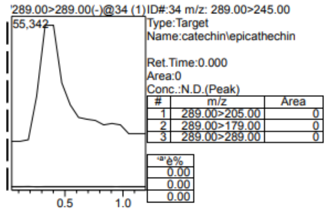
Gallocatechin\ Epigallocatechin	457.00>169.00<br>457.00>457.00	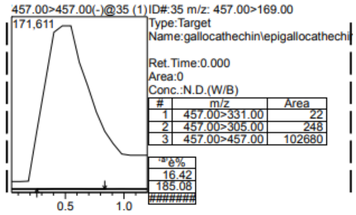	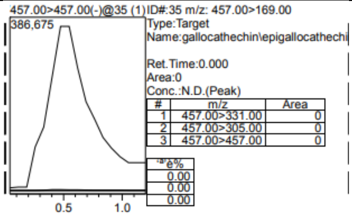
Rutin	609.00>301.00<br>609.00>609.00	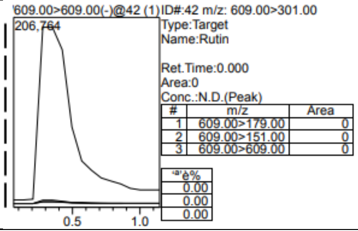	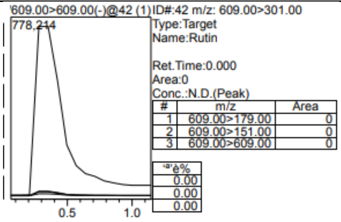

CSP Identified fragments according to the LC/MS-MS results
